# Ultrasound Detection of Nuchal Cord Tension in a Simulated Nuchal Umbilical Cord Model

**DOI:** 10.7759/cureus.115234

**Published:** 2026-08-26

**Authors:** Kimberly A Moyle, Amanda A Allshouse, Sonia G Parra, Nathan R Blue

**Affiliations:** 1 Obstetrics and Gynecology, Intermountain Health, Salt Lake City, USA; 2 Obstetrics and Gynecology, University of Utah Health, Salt Lake City, USA

**Keywords:** nuchal cord, prenatal ultrasound, simulation, stillbirth, umbilical cord tension

## Abstract

Background and objective

Ultrasound assessment of nuchal cord tightness may help identify fetuses at increased risk of cord accidents. Our objective was to assess ultrasound detection of simulated nuchal cord tension.

Methods

This was an ex vivo pilot study of human umbilical cords harvested from discarded placentas. The simulator consisted of a plastic bucket with a 5.1-cm-diameter cylindrical crossbar to simulate a fetal neck, filled with water and covered with a membrane. Using ultrasound, we measured deformation of the wrapped cord loop at 0, 2, 5, 7, and 10 lb of tension, using each cord’s off-tension free loop as a control. We used linear mixed-effects modeling to test the association of cord deformation with tension, accounting for cord characteristics.

Results

We evaluated 30 cords (mean length, 29.5 cm). Increasing tension resulted in increasing cord deformation between 0 and 5 lb, with no further deformation above 5 lb. The greatest deformation occurred between 0 and 2 lb, which was greater than the deformation occurring between 2 and 5 lb (p = 0.002). Cord length, days from delivery, and thickness were not associated with deformation.

Conclusions

In this proof-of-concept study, we detected nuchal loop tension using ultrasound in a simulated nuchal cord model, with the greatest deformation occurring between 0 and 2 lb.

## Introduction

Stillbirth is a common and catastrophic obstetric complication, occurring in 1 in 160 births in the United States [1]. Up to 20% of stillbirths may be attributable to cord accidents, wherein acute or chronic compression of the umbilical cord leads to hypoxia and fetal death [2]. These stillbirths are uniquely challenging because there are no clinically useful risk stratification tools to identify fetuses at risk of stillbirth from a cord accident before birth [2].

Nuchal cord occurs in up to 25% of deliveries and has been associated with intrapartum fetal distress and newborn asphyxia, but the association is weak and has not been consistently replicated [3-5]. More recently, investigators have suggested that prenatal ultrasound can be used to measure the tightness of the nuchal cord [6]. Assessment of nuchal cord tension may represent an opportunity to identify fetuses at risk of a cord accident.

Past in vitro umbilical cord models have studied the biomechanics of umbilical cords with external compression and demonstrated increased flow resistance with flattening of the umbilical vein [7]. If tension on a nuchal cord causes umbilical cord deformation in a predictable fashion, then it may be possible to use prenatal ultrasound to identify fetuses at greatest risk for flow disruption and subsequent stillbirth. However, it is unknown whether tension-associated cord deformation is detectable by ultrasound. The primary objective of this pilot proof-of-concept study was to determine whether ultrasound-measured deformation of an ex vivo human umbilical cord loop changes in response to controlled simulated nuchal cord tension. Secondary exploratory objectives were to characterize the relationship between increasing applied tension and cord deformation, assess whether cord characteristics influenced deformation, and evaluate whether deformation persisted after release of tension. We hypothesize that ultrasound will detect human umbilical cord deformation when increasing tension is applied in an ex vivo nuchal cord simulation model.

This work was presented as an abstract at the Society for Maternal-Fetal Medicine (SMFM) 42nd Annual Pregnancy Meeting, held virtually from January 31 to February 5, 2022.

## Materials and methods

We performed a pilot, proof-of-concept study utilizing ex vivo ultrasound measurements of human umbilical cord segments to explore the utility of prenatal ultrasound to detect nuchal cord tension. This study protocol was reviewed by our institutional review board (IRB) and determined to be exempt from IRB oversight as non-human subjects research. Deidentified cord segments were harvested from discarded placentas between 0 and 13 days after delivery without regard to mode of delivery or the use of delayed cord clamping, which is standard practice at our institution. Cord segments were excluded if the length was insufficient for use in the simulation model (<20 cm) or if there was an indication for placental histopathologic evaluation. After delivery, cords were stored still attached to the placenta in sealed plastic buckets prior to collection for use in the study. Although hydration was not specifically assessed or maintained, the airtight buckets and attached placentas prevented any visible desiccation. Umbilical cord clips were fastened to each end of the segment. An effort was made to retain as much cord blood as possible within the cord segment.

Prior to the simulator protocol, cord features were recorded. Cord segment length was measured and recorded. Based on visual inspection of the proportion of Wharton’s jelly to the cord diameter, each cord’s thickness was designated as thin, moderate, or thick. Cord thickness was based on subjective visual assessment using the following descriptions. Cords with no visible or very little Wharton’s jelly, where the external contours of all three vessels were easily visible, were described as thin. Cords with a moderate amount of Wharton’s jelly, the typical finding at delivery, were described as having moderate thickness. Cords in which the contour of the vessels was not externally visible and the majority of the cord volume appeared to be Wharton’s jelly were described as thick. Days from delivery were recorded for each cord.

**Figure 1:**
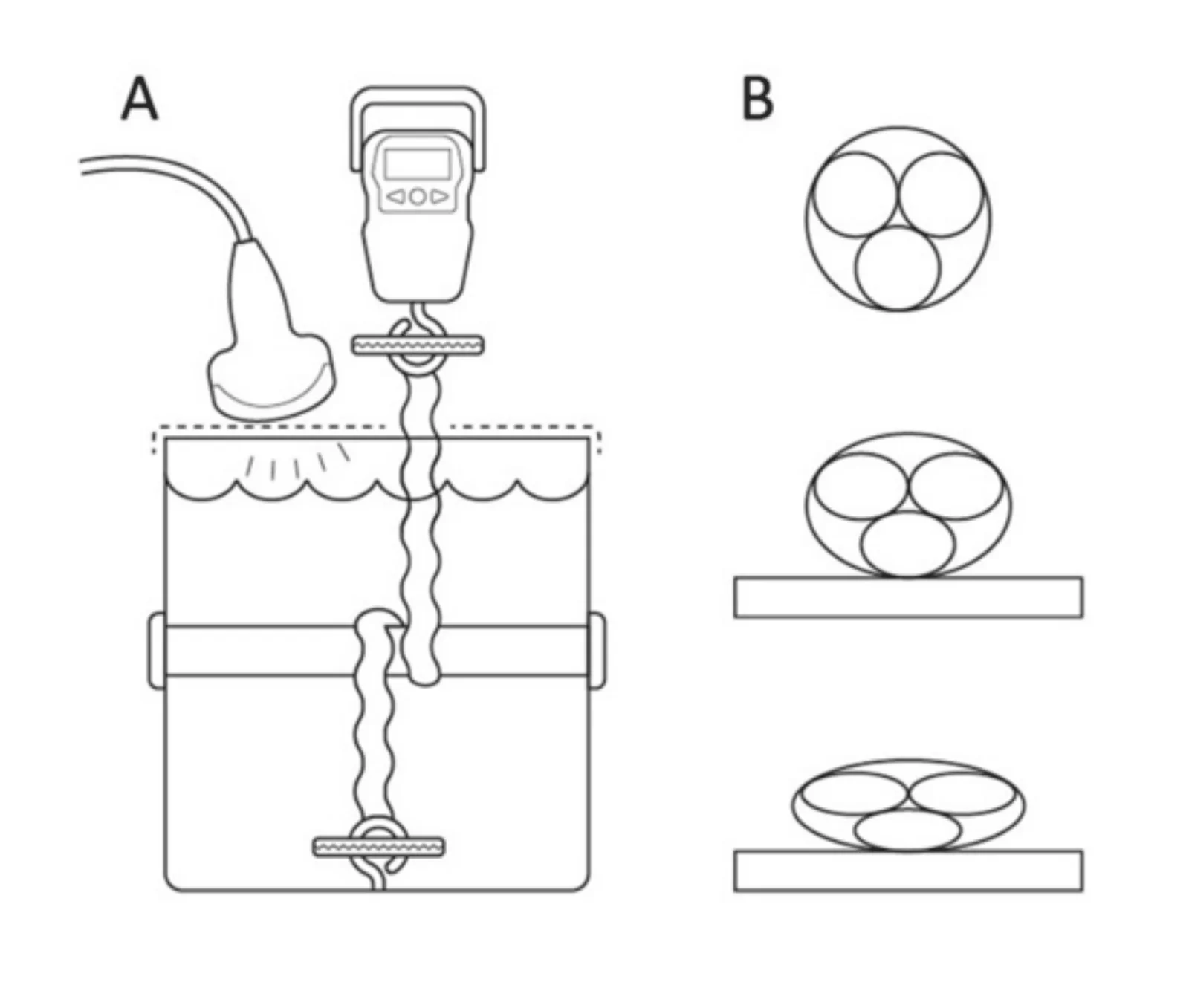
Schematic of the nuchal cord simulator and hypothesized progressive umbilical cord deformation with increasing tension. (A) Schematic of the nuchal cord simulator. (B) Illustration of cord appearance on ultrasound with increasing tension. This figure was created using Adobe Illustrator version 26.5 (Adobe Inc., San Jose, CA, USA).

The nuchal cord simulator consisted of a rectangular plastic container with a 5.1-cm-diameter cylindrical crossbar. A 2-cm metal loop was affixed to the base of the container. The container was filled with water and covered with a transparent membrane. One umbilical cord clip was fastened to the metal loop at the base of the container in a “toggle clasp” fashion. The cord was looped around the crossbar and then attached to a tension scale above the container. The simulation model is illustrated in Figure 1A.

We used a Samsung HERA W10 machine (Samsung Medison, Inc., Seoul, South Korea) and a 3-10 MHz curved-array probe to measure perpendicular cord diameters, height (H), and width (W) of a cross-section of a nonlooped, tension-free portion of the cord. The H/W ratio of this segment was used as the baseline dimension of each cord such that each cord served as its own control. The cord was visualized in the sonographic plane in which the crossbar appeared perpendicular to the angle of insonation at the center of the curvilinear transducer and with a true cross-section of the cord, where the lateral measurement was as narrow as possible. The cross-sectional height and width of the wrapped portion of the cord in closest proximity to the transducer were then measured (Figure 1). This measurement was taken at the point where the cord passed over the top of the crossbar. Diameters were measured and recorded with no applied tension initially and then repeated with increasing amounts of tension manually applied to the free end of the cord, measured in lb: 0, 2, 5, 7, and 10, or until the cord segment avulsed (Figure 1B). Once a cord was avulsed, no further cord deformation measurements were taken. Tension was applied by hand using the tension scale and slowly increased until the prespecified tension threshold was achieved. This level of tension was then maintained while the measurements were taken. Measurements were taken once at each level of tension, so intraobserver variability was not assessed. Once measurements were taken, additional tension was slowly applied until the next measurement threshold was reached. This sequence continued until a maximum tension of 10 lb was reached or until the cord segment avulsed. The tension scale was precalibrated and was not adjusted during the course of the experiment. Ultrasound settings were unchanged for the duration of the cord measurement process. For consistency, the same observer performed all ultrasound measurements. The ratio of the measured cross-sectional diameters, H/W, or “roundness,” was calculated for all measurements.

The primary outcome was cord “deformation,” defined as the ratio of the cross-sectional diameters of the wrapped loop of cord, normalized to the same cord’s cross-sectional diameter ratio of the off-tension free loop. This was computed as a ratio of ratios: (H/W of the wrapped loop):(H/W of the off-tension free loop). A deformation value close to one means the wrapped loop is similarly round to the same cord’s off-tension free loop and so represents little or no deformation, whereas a deformation value closer to zero means the wrapped loop is much less round than the off-tension free loop, representing more deformation.

We describe cords with mean length and median age and report the Pearson correlation and 95% CI between length and age and cord deformation. Using cross-sectional methods, the difference in deformation between 0 lb and 2 lb of tension was compared with the difference between 2 lb and 5 lb of tension with a paired t-test. To utilize the repeated measurements on the same cord, we used linear mixed-effects modeling to account for within-cord similarities and estimate the difference in deformation with increasing tension. We evaluated tension as a linear, quadratic, cubic, and quartic term in a linear mixed-effects regression model. We considered cord characteristics for inclusion in modeling: cord length, age (days since delivery), and thickness. We also assessed interactions between these cord characteristics and tension. Deformation was analyzed on the log scale, with results back-transformed and reported as geometric means and 95% CIs. Estimates were calculated using linear mixed-effects regression modeling, with first-, second-, and third-order terms for tension.

Based on informal observations during initial data collection, we began measuring deformation of the off-tension wrapped loop after tension was released to determine if induced cord deformation persisted or resolved. We compared the roundness after tension release to baseline using a paired t-test.

Two-sided p-values <0.05 were considered statistically significant. Analyses were performed using SAS software version 9.4 (SAS Institute Inc., Cary, NC, USA).

## Results

We evaluated 30 cords: mean length, 29.5 cm (SD, 7.4 cm); the majority were of moderate thickness (67%); and median cord age was 2 days (IQR, 1-3 days) (Table 1).

**Table 1:** Umbilical cord segment characteristics. Data are expressed as n (%) except where otherwise specified.

Characteristic	N = 30 cords
Thickness	
Thin	7 (23.3)
Moderate	20 (66.7)
Thick	3 (10.0)
Age (days from delivery)	
0	2 (6.7)
1	9 (30.0)
2	8 (26.7)
3	4 (13.3)
4	4 (13.3)
13	3 (10.0)
Median (P25, P75)	2 (1, 3)
Length (cm)	
Mean ± SD	29.5 ± 7.4

Baseline cord roundness was negligibly correlated with cord age (corr = 0.05, 95% CI, -0.3 to 0.4; p = 0.79) and weakly correlated with cord length (corr = -0.34, 95% CI, -0.6 to 0.4; p = 0.08); neither was significant.

**Figure 2:**
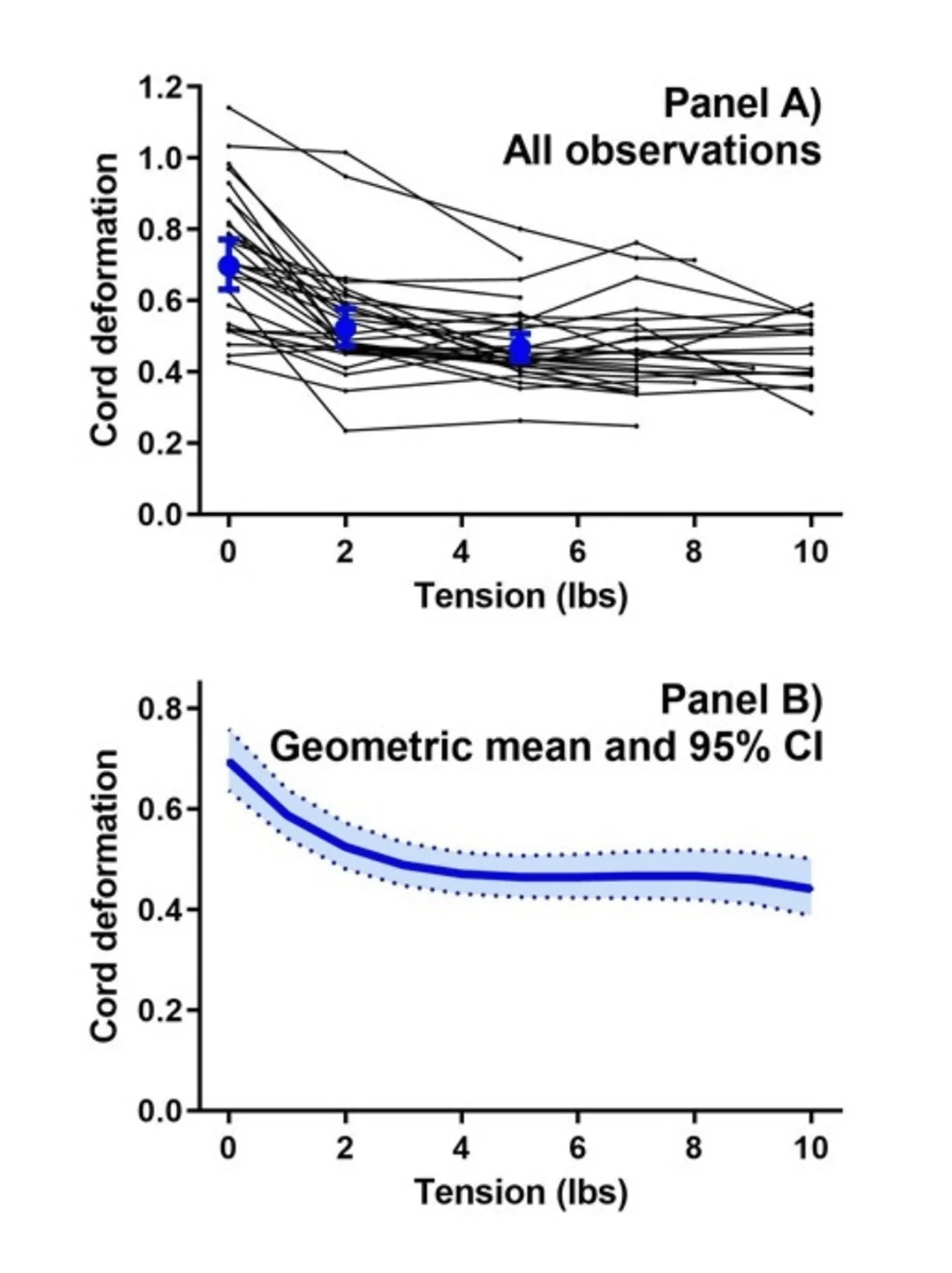
Umbilical cord deformation with increasing tension. (A) All individual observations plotted, with geometric mean and 95% CI plotted at 0, 2, and 5 lb. (B) Geometric mean and 95% CI from linear mixed-effects regression model, with first-, second-, and third-order terms for tension. Cord deformation is defined as the height-to-width ratio (roundness) of the wrapped loop to the height-to-width ratio of its off-tension free loop, where a decreasing value represents increasing deformation. For panel A, a significant change in deformation occurred between 0 and 2 lb (p < 0.001) and between 2 and 5 lb (p = 0.002). The deformation between 0 and 2 lb was significantly greater than that between 2 and 5 lb (p < 0.001). This figure was created using GraphPad Prism version 9.1.2 for Windows (GraphPad Software, La Jolla, CA, USA).

The trend in deformation with increasing tension is presented for all cords (Figure 2A).

We observed that between 0 and 5 lb of tension, there was an initial steep increase in deformation that leveled off after 5 lb. Mean deformation decreased from 0.70 (95% CI, 0.63-0.77) at zero tension to 0.52 (95% CI, 0.47-0.58) at 2 lb and 0.47 (95% CI, 0.43-0.51) at 5 lb. Both the changes between 0 and 2 lb (p < 0.001) and between 2 and 5 lb (p = 0.002) were significant, with the change from 0 to 2 lb being significantly greater than the decline from 2 to 5 lb (p < 0.001).

In a mixed-effects linear regression model, cord length, age, and qualitative thickness were evaluated for inclusion in the model but were not significantly associated with the outcome. A final model included an intercept and linear, quadratic, and cubic terms for pounds and demonstrated a similar shape to the unadjusted means (Figure 2B).

We initially attempted post-tension measurements following release of maximum applied tension, but most cords avulsed before achieving 10 lb. We therefore began measuring deformation after applying only 2 lb of tension. Among these cords (n = 12), mean deformation after release of tension (0.61, 95% CI, 0.51-0.74) was lower than baseline (0.70, 95% CI, 0.60-0.81; p = 0.002), suggesting partial persistence of deformation.

## Discussion

In this pilot study utilizing human umbilical cord segments in a simulated nuchal cord model, cord tension was detectable as deformation of the wrapped loop compared with the off-tension free loop. A positive relationship between cord deformation and the amount of applied tension was demonstrated, with the greatest change occurring between 0 and 2 lb of tension. Some degree of deformation persisted after release of the lowest applied level of tension, suggesting that induced deformation may persist longer than the application of tension. As we only measured post-tension deformation immediately following tension release, it is not known whether the deformation eventually resolves.

Very little has been published on ultrasound assessment of nuchal cord tightness. Zhao et al. evaluated ultrasound measurement of nuchal cord roundness using the ratio of cross-sectional diameters and its association with fetal arterial Doppler flow measurements [6]. They found a correlation between nuchal cord roundness and changes in fetal umbilical and renal artery flow but no significant association with fetal distress or cesarean delivery rates. Although a correlation with adverse fetal outcomes was not identified, the change in arterial flow characteristics suggests that nuchal cord tightness may impact fetal hemodynamics. This hypothesis is further supported by the findings of several investigations of umbilical cord flow in simulation models. One study found that umbilical veins become occluded with as little as 0.6-1.1 lb of tension in perfusion simulations of nuchal cords and true knots [8,9]. In an in vitro model testing the effect of external compression on umbilical cord venous flow, Pennati et al. found that umbilical vein flow was affected once compression reduced the cord diameter by 30-50%, which is similar to the degree of deformation we measured at 2 lb of tension [7]. Taken together, this prior work suggests that the lowest level of tension we assessed could plausibly impact cord venous flow and therefore fetal hemodynamics.

The strengths of our study include the use of human umbilical cord segments, direct ultrasound measurement of cord deformation, and direct control over the amount of applied tension. The change in roundness of each wrapped cord was normalized to its own off-tension free loop, a technique that can be adapted for in vivo use. All measurements were performed by the same observer for consistency. Limitations include our study’s small sample size and simple design of the simulator. We utilized a simple ex vivo model and therefore are not able to simulate other parameters that can affect fetal outcomes, such as fetal movement, amniotic fluid dynamics, fetal neck anatomy, vascular perfusion, fetal blood pressure, or real-time physiologic adaptation. The variability in cord thickness and testing several days after delivery may introduce a difference in deformation measurements that we have not yet identified. Furthermore, the lack of active flow through the umbilical vessels due to the closed ends of the cord segments may have affected how the cord flattens with tension. However, the closed cord ends would likely bias the results toward the null, given that the closed vessels should be less compressible than open vessels, suggesting that cord deformation may be even more prominent than our findings show. While having a single observer allowed for consistency in the simulation, further studies will be needed to determine interobserver reproducibility, intraobserver reliability, and the feasibility of detecting in vivo umbilical cord deformation during routine prenatal scanning.

## Conclusions

Our study was designed as a pilot, “proof-of-concept” study to assess whether ultrasound-measured deformation of an ex vivo human umbilical cord loop changes in response to controlled simulated nuchal cord tension. We demonstrated that increasing tension in simulated nuchal loops is detectable with increasing deformation and that the majority of deformation occurred at the lowest level of tension we measured. However, the relevance and utility of these findings remain unclear, as it is unknown how much tension plausibly occurs in vivo and whether ultrasound can detect the level of tension at which early fetal hemodynamic changes occur. Future work is needed to further characterize the relationship between increasing applied tension and cord deformation, including whether lesser degrees of tension induce ultrasound-detectable deformation; assess whether cord characteristics influence deformation; evaluate whether deformation persists after release of tension; and determine the effect of tension on flow in the umbilical vessels. We conclude that ultrasound can detect nuchal cord tension in a simulated ex vivo nuchal cord model and plan additional studies focusing on deformation associated with less than 2 lb of tension and correlation with cord vessel flow dynamics.
